# Innovative Wound Healing Hydrogel Containing Chicken Feather Keratin and Soy Isoflavone Genistein: In Vivo Studies

**DOI:** 10.3390/gels9060462

**Published:** 2023-06-05

**Authors:** Nilesh M. Mahajan, Kalyani Wanaskar, Nemat Ali, Debarshi Kar Mahapatra, Muzaffar Iqbal, Abid R. Bhat, Mohammed Kaleem

**Affiliations:** 1 Department of Pharmaceutics, Dadasaheb Balpande College of Pharmacy, Rashtrasant Tukadoji Maharaj Nagpur University, Nagpur 440037, Maharashtra, India; nmmahajan78@gmail.com (N.M.M.); wanaskarkalyani@gmail.com (K.W.); 2 Department of Pharmacology & Toxicology, College of Pharmacy, King Saud University, P.O. Box 55760, Riyadh 11451, Saudi Arabia; 3 Department of Pharmaceutical Chemistry, Dadasaheb Balpande College of Pharmacy, Nagpur 440037, Maharashtra, India; dkmbsp@gmail.com; 4 Department of Pharmaceutical Chemistry, College of Pharmacy, King Saud University, Riyadh 11451, Saudi Arabia; muziqbal@ksu.edu.sa; 5 Department of Emergency Medicine, University of Maryland School of Medicine, Baltimore, MD 21201, USA; 6 Department of Pharmacology, Dadasaheb Balpande College of Pharmacy, Rashtrasant Tukadoji Maharaj Nagpur University, Nagpur 440037, Maharashtra, India

**Keywords:** chicken feather, keratin, genistein, hydrogel, wound healing, ELISA, qRT-PCR

## Abstract

The current study was performed to isolate keratin from chicken feathers with an intention to develop a keratin–genistein wound-healing hydrogel, along with its in vivo analysis. Pre-formulation aspects were analysed by using FTIR; SEM; HPTLC, while gel was characterized for gel strength, viscosity, spreadability, drug content, etc. Additionally, an in vivo study along with biochemical factors against pro-inflammatory factors and histopathological studies were conducted to determine possible wound-healing and anti-inflammatory effects. Pre-formulation studies revealed the presence of amide bonds with region of dense fibrous keratin and an internal porous network in extracted keratin, which corresponds with standard keratin. Evaluation of optimised keratin–genistein hydrogel indicated the development of neutral, non-sticky hydrogel which spread evenly on the skin. In vivo studies in rats indicate higher degrees of wound-healing in combined hydrogel (94.65%) for a duration of 14 days as compared to an individual hydrogel formulation with the development of the epidermis and excessive proliferation of fibrous connective tissue indicating wound repair. Furthermore, the hydrogel inhibited the overexpression of IL-6 gene along with other pro-inflammatory factors, indicating its anti-inflammatory effects. In order to find out the possibility of closure of wounds and anti-inflammatory properties of the novel product, an in vivo investigation into the healing of wounds in laboratory animals was carried out through biochemical (ELISA and qRT-PCR) analyses against inflammatory markers (IL-2, IL-6, IL-1, IL-10, and COX-2) and histopathological (liver, skin, and the kidneys) investigations. Based on the results, we conclude that keratin–genistein hydrogel is a promising therapeutic molecule for the management of wound repair.

## 1. Introduction

For healthcare systems, skin wounds represent a significant burden. Their high rates of morbidity chronification, and relapse, pose a significant strain on healthcare-related aspects of living, elevating both individual and societal expenses as well as posing a problem for healthcare systems across the globe [1,2,3]. Based on the ageing of the population and a rise in the rates of diabetes, overweight, and heart disease, skin wounds and their potential to become chronic will likely grow more common [2,4]. As a result, managing wounds is a major issue that is becoming more and more prevalent globally [4,5]. As such, it is crucial to develop new products that can accelerate the healing process.

When the integrity of any tissue is damaged, a wound develops (such as when the skin or a muscle becomes damaged, a bone is fractured, or burns occur) [6]. A wound could occur from a fall, surgery, an infection, circumstances, or another event [7]. Several antioxidant genes, including catalase (CAT) and glutathione peroxide (GPx), are expressed more frequently during the natural healing of wounds, indicating a possible function for free-radical-scavenging enzymes [8].

In order to promote cellular growth and cell-guided tissue development, the protein layer has a distinct three-dimensional architecture. Hair, nails, and other areas of the skin contain large amounts of the naturally occurring protein keratin [9]. In comparison with the other proteins, it contains greater levels of cysteine [10]. The protein is hard, resilient, and lighter because the sulphur atoms in the remnants of cysteine frequently interconnect with each other [11]. Chicken feathers can serve as a good source of protein due to their elevated keratin protein level [12].

Genistein, which is extracted from soybean, is used as an inhibitor of epidermal growth factor (EGF)-induced proliferation [11,13]. It promotes the synthesis of collagen, which is important for preserving the equilibrium of the skin as well as accelerating wound repair [12]. In human dermal fibroblasts, genistein has a direct influence on the signalling pathways which govern collagen production [14]. T-BHP (t-butylhydroperoxide) induces collagen production in the presence of oxidative stress [15]. Because of its antioxidant effects, this phytochemical guards human dermal fibroblasts from oxidative-stress-related production of collagen suppression [16]. Through changing the inflammatory reaction, it further modulates the healing of wounds [17,18]. Through enhancing antioxidant activity while regulating the expression of cytokines that are pro-inflammatory in the initial stages of wound repair, genistein supplements decrease the risk of oxidative stress [19]. Without proper antioxidant activities, wound healing might be delayed, or severe tissue damage can occur [20]. Isoflavone (genistein) in soybean has been reported to have potent antioxidant properties and also possess pharmaceutical potency with oestrogen-like activity [21].

The current study was performed to isolate keratin protein from chicken feathers with the intention to develop a keratin–genistein-combination-based wound-healing hydrogel. Since hydrogel polymer material can absorb and hold a greater amount of exudates, it was rationally chosen for this study. The pre-formulation aspects, such as protein content, drug–excipient interactions, etc., were determined through standard methods and sophisticated instruments (UV, FT-IR, SEM, and HPTLC). The prepared gel was evaluated comprehensively with respect to the physicochemical parameter (gel strength, viscosity, pH, spreadability, drug content, etc.). An in vivo wound-healing study in experimental animals followed by biochemical assays (ELISA and qRT-PCR) against pro-inflammatory factors (IL-2, IL-6, IL-1β, IL-10, and COX-2) and histopathological (liver, skin, and kidney) studies were performed to determine the possible wound closure and anti-inflammatory effects of the innovative product.

## 2. Results and Discussion

### 2.1. Characterisation of Keratin

#### 2.1.1. Protein Content

The protein content as determined by the Kjeldahl method showed 86.89% *w*/*w* of protein content from 500 mg of keratin sample. This was found to be a good percentage of protein content available in the keratin sample, as it was expected to contain above 80% *w*/*w* [22].

#### 2.1.2. Amino Acid Profiling

As per the literature, total amino acid contents must exceed 70% of the keratin obtained from the chicken feathers. The keratin sample displayed the presence of aspartic acid, alanine, glutamic acid, serine, threonine, and tyrosine in the range of 0.36 to 12.1 g/100 g. These values corresponded with the reference range of standard keratin (Table 1). Serine, glutamic acid, and aspartic acid were found in higher concentrations than tyrosine, alanine, and threonine [16].

#### 2.1.3. Fourier-Transform Infrared (FT-IR) Spectroscopy

The absorption bands that were observed in the keratin sample are mainly because of the amide bonds (-CONH-). Among nine amide bands, five were prominently observed, viz., amide-I, II, III, A and B. The signal at approximately 3300 cm^−1^ corresponds to amide A. The Amide B band has been reported and appeared within 3056 to 3075 cm^−1^. The strong peaks at 1654 cm^−1^ were assigned to amide-I, which is closely related to the carbonyl groups. Amide–II was observed within 1480–1580 cm^−1^, which is related to symmetric bending vibrations of –NH and –CN. The bands of approximately 1230–1240 cm^−1^ were observed and associated with β-sheet of amide-III. All the characteristic bands that were observed in the FT-IR spectrum of extracted keratin were indicative of its similar secondary structure as that of the standard [23].

The FT-IR spectrum of pure genistein shows a broad band at 3464.15 cm^−1^, corresponding to the phenolic–OH group, and carbonyl stretching vibrations were observed at 1625.99 cm^−1^. The C-H aromatic band was observed at 3041.74 cm^−1^, whereas the band of carbonyl functionality C=O was observed at 1159.22, and that of the C-O stretching band was seen at 1022.27 cm^−1^. Aromatic C=C stretching bands were observed in the range of 1600–1400 cm^−1^. All the characteristic bands were observed in the FT-IR spectrum of pure genistein, confirming its identity and purity [24,25]

**Figure 1:**
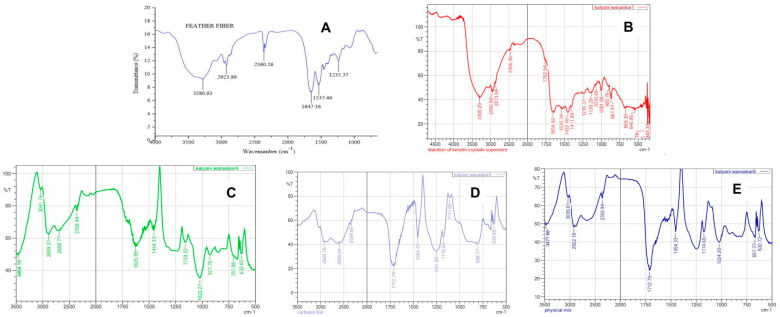
FT-IR spectra of (**A**) reference keratin; (**B**) extracted keratin; (**C**) genistein; (**D**) Carbopol 934; and (**E**) physical mixture.

The FT-IR spectrum of the physical mixture of keratin and genistein with Carbopol 940 and other excipients was found to retain all major peaks shown in pure keratin and genistein. It revealed no interaction of the drug component with the excipients, and thus, the formulation is considered stable and compatible, as shown in Figure 1.

#### 2.1.4. High-Performance Thin Layer Chromatography (HPTLC)

**Figure 2:**
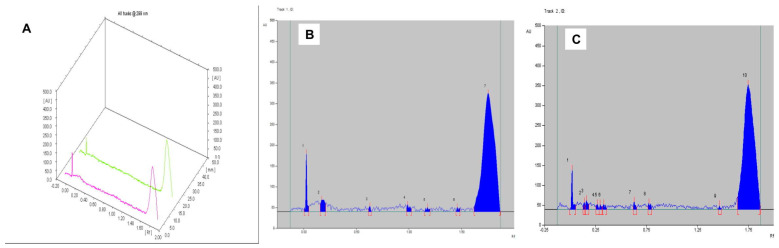
HPTLC of (**A**) Comparative chromatograms of extracted keratin and keratin gel; (**B**) extracted keratin; and (**C**) keratin gel.

The solvent system butanol: acetic acid: water in the ratio of 6.5:3.5:1 presented Rf value of 0.63 for the extracted keratin, which was also found in the keratin gel with an alike Rf value (Table 2). The chromatogram of the keratin gel produced the related characteristic peaks with a% corresponding area of 0.57 and 0.95, respectively (Figure 2). The most abundant constituents of keratin were found in the final formulation, which represents the intactness of the keratin in the gel.

#### 2.1.5. Scanning Electron Microscopy (SEM)

**Figure 3:**
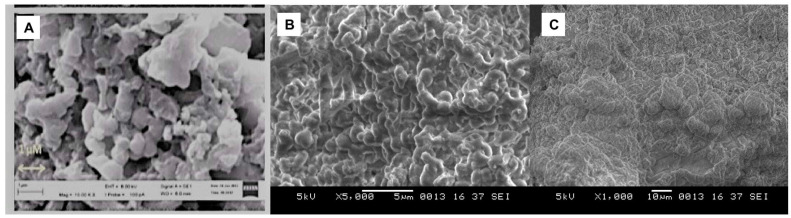
Microphotograph of (**A**) reference keratin; (**B**) extracted keratin at 5000×; and (**C**) extracted keratin at 1000×.

The morphology of the keratin crystals at both 5000× (Figure 3B) and 1000× (Figure 3C) was investigated via SEM analysis. The microphotographs presented a region of dense fibrous keratin with an internal porous network (Figure 3A).

### 2.2. Characterisation of Genistein

#### High-Performance Thin Layer Chromatography (HPTLC)

**Figure 4:**
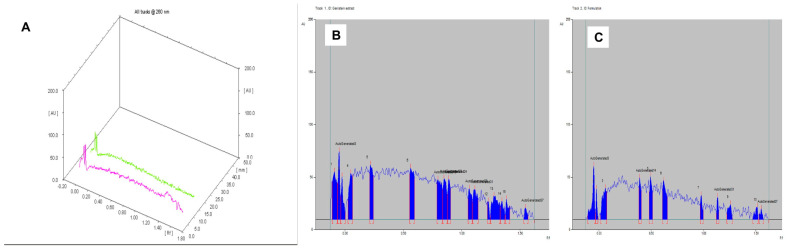
HPTLC of (**A**) comparative chromatograms of extracted genistein and genistein gel; (**B**) extracted genistein; and (**C**) genistein gel.

The solvent system chloroform: methanol in the ratio of 10:1 presented an Rf value of 0.49, which was also found in genistein gel (Rf = 0.50) (Table 2). The chromatogram of the genistein gel produced the related characteristic peaks with a % corresponding area of 11.03 and 13.34, respectively (Figure 4). The most abundant constituents of genistein were found in the final formulation, which represents the intactness of the genistein in the gel.

### 2.3. Characterisation of Gel Formulations

#### 2.3.1. General Characterisation

The initial batches of the gel base were characterised for their consistency, pH, viscosity, and spreadability. Based on the study base formula, F1 was found to be difficult and not acceptable. The viscosity was also found to be quite high. In contrast, the F2 showed a slightly acidic nature and sticky content. The formulation F3 represented a non-sticky consistency, which favoured the ease of its application to the skin surface, and the pH was found to be neutral. Furthermore, the drug content study was performed to determine the content of the drug that was present in the optimised formulation. An optimised gel formulation was found to be a satisfactory drug content that ranges from 91 to 100% of keratin and genistein extract (Table 3).

#### 2.3.2. High-Performance Thin Layer Chromatography (HPTLC)

**Figure 5:**
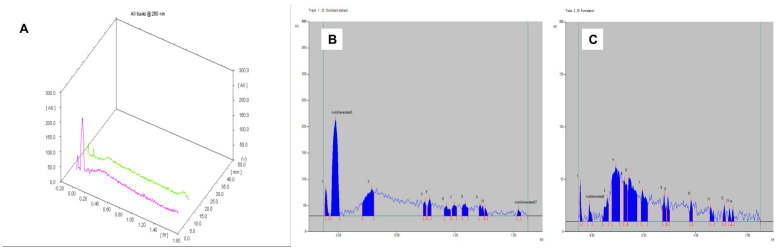
HPTLC of (**A**) comparative chromatograms of extracted keratin, genistein, and keratin–genistein combination gel; (**B**) extracted keratin, genistein; and (**C**) keratin–genistein gel under mobile phase system chloroform: methanol (10:1 *v*/*v*).

The most abundant constituents of keratin and genistein were found in the final formulation. The mobile phase system comprised butanol: acetic acid: water (6.5:3.5:1 *v*/*v*/*v*); the keratin extract showed an Rf value of 0.58, which was also found in the keratin–genistein gel (Rf value of 0.51) (Table 2). The most abundant constituents of keratin and genistein were found in the final formulation. The mobile phase system comprised chloroform: methanol (10:1 *v*/*v*); the genistein extract demonstrated an Rf value of 0.74, which was equivalent to the keratin–genistein gel (Rf value of 0.70) (Table 2). However, only genistein runs in this solvent system. The chromatogram of keratin, genistein gel revealed similar characteristic peaks to that of genistein, which represented the intactness of the genistein in the gel formulation. The corresponding % area was found to be about the same (3.68 and 4.87) (Figure 5).

### 2.4. Stability Studies

The stability studies were carried out for the optimised formulation at 40 ± 2 °C temperature and 65 ± 5% RH for a period of 90 days [26]. The formulation presented good stability over the time regimen with no remarkable change in the pH, viscosity, and spreadability test profile (Table 4).

### 2.5. Wound-Healing Activity

**Figure 6:**
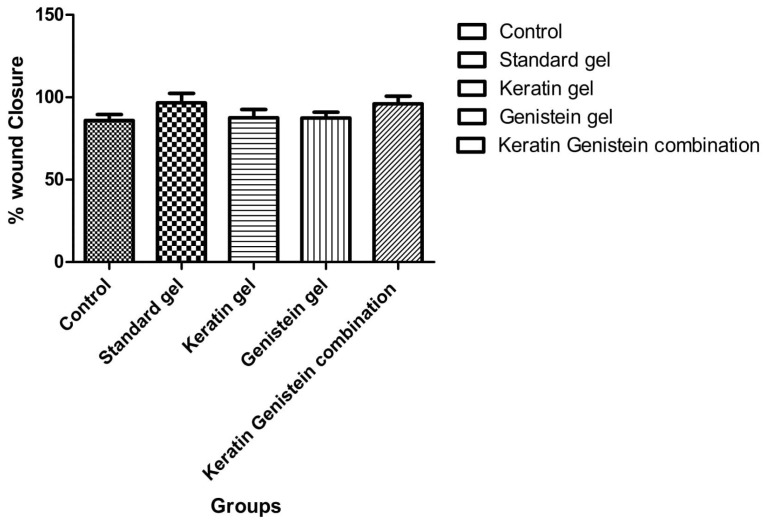
Percentage of wound closure after treatment with various gel formulations.

**Figure 7:**
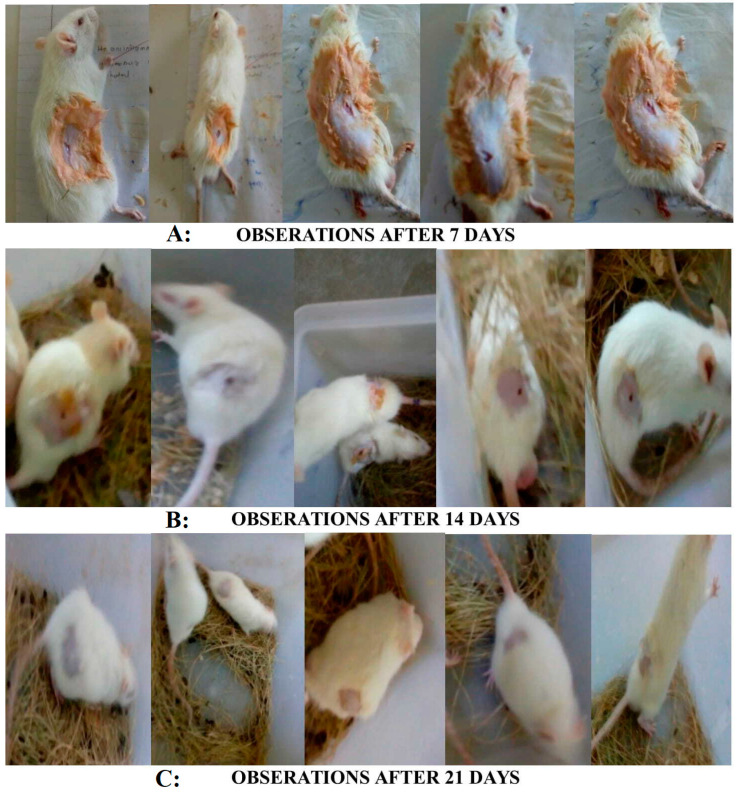
This figure indicates morphological studies of wound-healing activity of formulation after (**A**) 7 days; (**B**) 14 days; and (**C**) 21 days.

Keratin–genistein gel formulation showed higher degrees of wound healing (94.65%) for the duration of 14 days as compared to the keratin gel formulation (87.57%) and genistein (87.40%) gel formulation (Table 5). Furthermore, the keratin–genistein-combination gel formulation presented a comparable activity to that of the marketed hydrogel product (96.66%), as well as the control group (85.98%) (Figure 6). The stages of healing by the keratin–genistein-combination gel formulation in the experimental animal model are described in Figure 7. The present observations and potential roles of genistein in promoting wound healing were found to be comparable with the results stated in previous reports on early-stage cutaneous wound healing, refractory wound healing in type-1 diabetes, skin wound repair in an incision model, and wound healing in ovariectomised mice [27].

### 2.6. Histopathological Investigations

After 14 days, the wound-healing activity produces a normal architecture of the skin. As compared to the genistein gel and keratin gel, the keratin–genistein combination gel produced proper development of the epidermis, along with the proliferation of fibrous connective tissue and the growth of hair follicles. After 14 days, the normal functioning of the kidney and liver was studied, where wound healing does not produce any toxic effect. Genistein purposely solved the development of epidermal growth on the skin. The control sample of the skin showed failing to develop an epidermis and no proliferation of fibrous connective tissue. The keratin sample presented the development of epidermis and the proliferation of fibrous connective tissue, indicating wound healing. The genistein gel sample revealed the development of epidermis and excessive proliferation of fibrous connective tissue, demonstrating wound healing [23,28].

**Figure 8:**
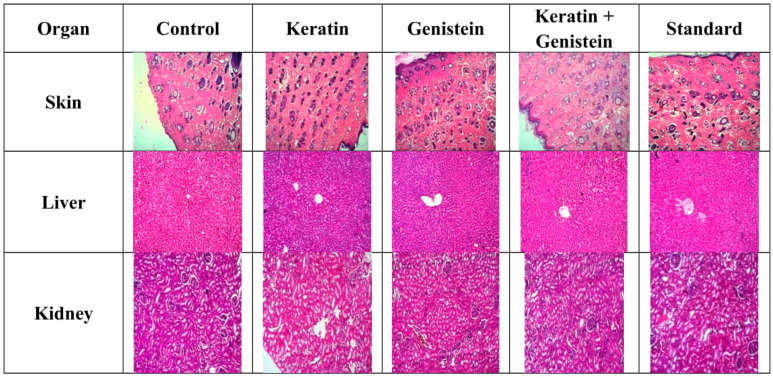
This figure shows that histopathological studies of skin, liver, and kidney.

The combination of keratin and genistein showed proper development of epidermis, along with the proliferation of fibrous connective tissue and growth of hair follicles. The standard sample displayed the best wound healing, with development of epidermis and proliferation of fibroblastic cells and hair follicles. The control sample of the liver revealed vacuolar degenerative changes in hepatocytes around the central vein. The keratin, the genistein, the standard, and the combination sample showed the normal architecture of hepatocytes around the central vein. The control sample of the kidney revealed the inflamed architecture of glomeruli and tubules. In contrast, keratin, genistein, standard, and the combination sample showed the normal architecture of glomeruli and tubules (Figure 8).

In the case of vital organ toxicity studies, the histological section of liver shows normal architecture of hepatocytes around the central vein as compared to vacuolar degenerative changes in positive control group. Kidney histology shows the normal architecture of glomeruli and tubules. This result proved the safety of keratin and genistein for medicinal use.

### 2.7. Biochemical Investigations

**Figure 9:**
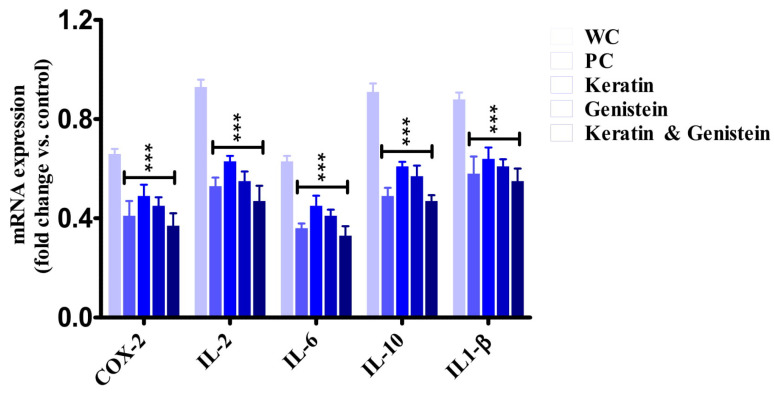
This figure shows mRNA expression levels of COX-2, IL-2, IL-6, IL-10, and IL1-β in the liver through qRT-PCR. [WC: Working Control; PC: Positive Control; *** represents the level of significance in comparision with working control].

The biochemical investigations through ELISA and qRT-PCR studies strongly advocated that the keratin–genistein combination gel-formulation-treated group produced a comparatively high impact over the IL-6 expression via the downregulation of the over-expressed IL-6 gene in contrast to the working control group (Figure 9). In comparison with other pro-inflammatory factors such as COX-2, IL-2, IL-10, and IL-1β expressed in the liver tissues, genistein and keratin, both in combination, have the capacity to regulate the higher expression of mRNA of IL-6 in inflamed liver tissues and, therefore, play an imperative role in the suppression of inflammation [29,30]. The exploration of the underlying mechanism(s) supported our hypothesis that the combination markedly normalised (*p* < 0.001) the upregulation of the IL-6 gene and exerted an anti-inflammatory effect by modulating the molecular targets (Table 6). The efficacy of the gel combination was observed to be quite comparable with the positive control (marketed standard formulation) at an analogous dose.

The inflammation-reducing potential of genistein by reducing the levels of cytokines (IL-1β, IL-2, IL-6, and IL-10) was found to have complied with the previous reports on the protective actions of this natural product in gut inflammation, inflammation that is related to colitis, TNF-α-induced endothelial inflammation, and vascular inflammation [23,31].

## 3. Conclusions

A safe and effective keratin, genistein wound-healing combined gel formulation was successfully developed and comprehensively characterised through sophisticated analytical techniques. The major active components that were needed for the wound-healing activity were identified, along with other components, by using TLC, FTIR, and amino acid profiling techniques. The present investigation revealed that the fabrication of keratin gel showed excellent wound-healing properties, and with the combination of the inhibitor of EGF- induced proliferation, genistein showed noteworthy wound-closure activity. The fabricated formulations demonstrated good spreadability with a pH suitable for human skin application. The formulations also retained a good stability condition over a period of 90 days. The ability of the natural-component-containing gel product as an effective wound healer was confirmed via histopathological studies. From all the biochemical studies, it may be concluded that the novel keratin, genistein wound-healing gel effectually reduced the pro-inflammatory factors (IL-2, IL-6, IL-1β, IL-10, and COX-2), which led to acute lessening of inflammation. Comparing the gel to other commercially available formulations, its desirable formulation texture characteristics, in addition to its viscosity, pH, spreadability, and wound-healing function, contributed to the quality and stability attributes of the gel.

## 4. Material and Methods

### 4.1. Material

Keratin was successfully extracted from chicken feathers based on an existing method. Genistein was purchased from a local shop, namely, Green Heaven Lab Ltd., Nagpur, Maharashtra, India. Carbopol 934 was procured from Apex Drug House Ltd., Mumbai, India. LOBA Chemie Ltd., Mumbai, India, supplied analytical-grade methyl paraben and propyl paraben. Cremophor RH-40 was obtained from PIOMA Chemicals, Mumbai, India. The chemicals that were used in the study were of analytical grade and obtained in the highest pure form. Double-distilled water (Borosil, Mumbai, Maharashtra, India) was utilised for the experiment. The standard keratin was obtained from Sigma-Aldrich, St. Louis, MO, USA, with CAS No. 69430-36-0.

### 4.2. Extraction of Keratin from Chicken Feathers

Chicken feathers of a sufficient quantity were obtained from a nearby poultry farm and soaked in ether for a period of 24 h. The wet feathers were exposed to sunlight until they were completely dried. The dried feathers were further cut uniformly into small pieces and blended. The blended feathers were sealed carefully in a sealed plastic bag. In a 2 L conical flask, 0.5 M of sodium sulphide solution was prepared, and the weighed amounts of 50 g (Shimadzu BL-220H, Kyoto, Japan) of blended chicken feathers were added. The solution was stirred by a mechanical stirrer (Biotechniques BIPO5B, India) for the duration of 6 hrs at a temperature of 30 °C and the target pH range of 10–12. Furthermore, the solution was filtered off to acquire the supernatant liquid. The supernatant liquid was taken into a beaker and stirred. In a conical flask, ammonium sulphate solution (0.7 g/mL) was prepared in double-distilled water with continuous stirring until all particles were dissolved [32,33]. The solution was subsequently filtered, and the obtained filtrate was added drop wise to the previous breaker containing the feather filtrate solution in a ratio of 1:1 with fast stirring. The supernatant precipitates were collected separately. The collected solid particles were added to ethanol (100 mL), and a pH of 12 was maintained with a NaOH solution (0.1 N) with continuous stirring. Additionally, the solution was placed overnight on a mechanical shaker. Finally, the solution was filtered to procure the solid particles and the supernatant liquid. The solid particles were dissolved in 0.5 N NaOH solution. Ultimately, the liquid was collected, and the solid materials were discarded. The liquid sample was then dried, and the keratin crystals were collected [22].

### 4.3. Characterisation of Extracted Keratin

#### 4.3.1. Protein Content

The protein content of the keratin sample was estimated via the Kjeldahl digestion flask method, as reported in the standard literature [34]. Protein content was determined by multiplying nitrogen contents by conventional factor 6.25, as the average nitrogen (N) content of most of the proteins was found to be about 16%. The % total nitrogen content in the sample was determined from the following formula [32]:

% Total Nitrogen = (Blank titration−Sample titration) × 1.4 (constant factor for Nitrogen) × 2 × Exact normality of 0.1N NaOH solution

#### 4.3.2. Amino Acid Analysis

The amino acids present in the feather keratin were estimated quantitatively by using the post-column derivatisation method with Ninhydrin applications followed by an amino acid analyser. By using 6 N HCl, the keratin samples (n = 4) were hydrolysed in a glass tube. The hydrolysed amino acids were then characterised via reverse-phase HPLC (RP-HPLC) [35].

#### 4.3.3. Scanning Electron Microscopy (SEM)

The morphology of the extracted keratin sample was characterised by electron microscopy (Jeol JSM-6360A, Japan). The dried sample was sprinkled lightly over the aluminium stub (3–5 nm; 75 s; 40 W) connected with double adhesive tape. The sample underwent a gold coating to enhance the electrical conductance. The stub containing the gold-coated sample was scanned randomly, and at 10 kV acceleration voltage, the photomicrographs were recorded [32].

#### 4.3.4. Fourier-Transform Infrared (FT-IR) Spectroscopy

The Fourier-transform infrared (FT-IR) spectroscopic (Shimadzu IR-Affinity-1, Kyoto, Japan) investigation of keratin, genistein, Carbopol 934, and a physical mixture in the range of 4000 cm^−1^ to 400 cm^−1^ was studied to determine the inherent stability and compatibility of the formulation through various chemical groups. Any such changes perceived through the spectra are a clear reflection of some possible interactions between the drug and the polymer [36].

#### 4.3.5. High-Performance Thin Layer Chromatography (HPTLC)

##### (a) For Keratin

High-performance thin-layer chromatography (HPTLC) (Camag, North Carolina, USA) was performed for the keratin samples on 10 cm × 10 cm F_254_ HPTLC Si-60, diol, CN, NH2, and RP-18 plates. The sample and standard were spotted as bands of 5 mm width by using the automatic applicator Desaga AS 30 (Heidelberg, Germany). The samples were applied in the triplicate manner in the form of a band of 6 mm in the concentration of 10 μL. The distance between the tracks was kept to 10 mm at an application rate of 110 nL/s for the application of the bands. The plates were developed in a linear ascending mode at 85 mm in a chromatographic horizontal Teflon DS chamber (Chromdes, Lublin, Poland) that was previously saturated with some vapours of the mobile phase of butanol: acetic acid: water in the ratio of 4:1:1 *v*/*v*/*v*. The plates were developed after drying in the warm air streams and observed at 254 nm and 366 nm under a UV lamp. Densitometric scanning was performed by using the TLC scanner-III in the pre-absorbance/reflectance mode [37].

##### (b) For Genistein

In the present study, 10 cm × 10 cm F_254_ HPTLC Si 60, diol, and CN, NH2, and RP-18 plates were used for thin-layer chromatography (TLC). A Desaga AS 30 automatic applicator (Heidelberg, Germany) was used to identify standards and samples within 5 mm bands. In a chromatographic horizontal Teflon DS chamber (Chromdes, Lublin, Poland), which had been earlier saturated with mobile phase vapours, the plates were developed to an 85 mm thickness. The plates were examined at 254 nm and 366 nm under a UV light after being dried in a stream of hot air. On pre-coated TLC Plates (10 cm × 10 cm) and by using a concentration of 10 μL, the samples were used in triplicates in the shape of a band with a width of 6 mm via a Linomat-V sample applicator. A 110 nL/s rate of application was used to apply bands while maintaining a 10 mm space among the tracks. In a glass chamber with twin troughs that had been saturated with mobile phase, linear ascending progression was performed. By utilising the TLC scanner III in the pre-absorbance/reflectance mode, densitometric scanning was carried out. HPTLC-plates were used to hold the commercial and enzymatic genistein hydrolysates. Chloroform: methanol (10:1 *v*/*v*) was used for the development as long as the solvent front travelled to the highest point of the plate [33,37].

### 4.4. Characterisation of Genistein

A pre-formulation study of the drug moiety was performed to determine the authenticity of the drug and to estimate its associative properties that may influence formulation development. As per the methods that were comprehensively described in the previous section, the λmax of the compound through UV-Vis spectroscopy, FT-IR, and HPTLC were performed.

### 4.5. Formulation Development (Gels)

#### 4.5.1. Optimisation of Gel Base

A lack of adequate activity of antioxidants, tissue injury, or healing of wounds may both be impaired. In-process quality control (IPQC) is a vital phase in the formulation of keratin protein wound-healing gel. In order to ensure that the final goods remain uniform from run to run, efficient over a period of time, and risk-free to be utilised, certain particular tests are carried out at different stages of the process of production. The first step that is performed is to verify the unprocessed products to determine if they agree with the standards that have already been established. As a result, the pH, viscosity, and amount of drug of the test formulation were examined. Certain distinct quality control criteria, such as texture profile, must be properly taken into account in the production of a gel (a semisolid formulation), with the aim of increasing stability, elegance, and subsequent approval from customers. The 1% gel formulation was prepared by taking 1 g of Carbopol 934, which was further added to 100 mL of distilled water and hydrolysed for duration of 24 h.

#### 4.5.2. Formulation Development of 2% Keratin Gel

In total, 1 g of keratin was taken (powder form obtained through freeze-drying technique) and triturated with 3 mL of Cremophor RH-40 (as a solubiliser) in a mortar and pestle. Furthermore, 50 g of hydrated Carbopol 934 was added to the above content. The preservatives (propyl paraben and methyl paraben) were added to the required amount. The pH of the formulation was maintained by adding Triethanolamine to form the gel product (Table 7).

#### 4.5.3. Formulation Development of 1% Genistein Gel

In total, 50 g of hydrated Carbopol 934 was taken, and 0.5 g of genistein was added. The content was mixed vigorously with a stirrer, and in required quantities, the preservatives (propyl paraben and methyl paraben) were added. The pH of the product was maintained by using triethanolamine (Table 7).

#### 4.5.4. Formulation Development of Keratin–Genistein Combination Gel

In total, 0.5 g genistein was dissolved in 50 mL of hydrated Carbopol 934 (Sample 1). A total of 1 g keratin crystal was mixed with 3 mL of the solubiliser Cremophor RH-40 in a mortar and pestle (Sample 2). Sample 2 was added to sample 1, and preservatives (propyl paraben and methyl paraben) were added. The pH of the gel formulation was maintained by using Triethanolamine (Table 7).

### 4.6. Characterisation of the Gel Formulation

#### 4.6.1. Physical Evaluation

##### Gel Strength

The gel strength was tested by the gel strength apparatus in accordance with the method. The 100 mL measuring cylinder was filled with the gel formulations, and then the gel was loaded with a 50 g piston. The gel strength was determined from the time (in seconds) required to move the piston 5 cm down through the gel. In this case, it took more than 5 min to drop the apparatus into the gel strength, and it was described by the minimum weights that pushed the apparatus 5 cm down through the gel.

##### pH

The pH of the gel formulations was determined by utilising the digital type of pH meter (Global-pH-DPH-507, Delhi, India) in the calibrated range of 4 and 7. In total, 1 g of the gel was placed into double-distilled water, and both the reference electrode and the glass electrode were dipped completely into the formulations to achieve the pH of the products. The experiment was executed in a triplicate form.

##### Viscosity

The viscosity of the products was determined by using the Brookfield Viscometer (DV-E1 model, Middleboro, MA, USA) at 0.5 rpm and using the no. 64 spindle in the temperature range of 25 ± 1 °C. The experiment was carried out in a triplicate manner.

##### Spreadability

On the principle of slip–drag, the spreadability of the gel formulations was determined. The required amount of the formulation was placed on the ground slide, and the test material was prepared such that it was sandwiched by another glass slide which comprised a hook system. A pressure of 1 kg of mass was applied to the slides to remove the air entrapped between the thin films. The excess content of the formulation was swiped off from the edges of the slide, and the top slide was dragged with a force equivalent to 50 g intensity. The time required for the top slide to cover 7.5 cm distance was estimated and concluded from the following formula:

S = M × L/T

where S = Spreadability coefficient, M = weight applied, L = length moved by the glass slides, and T = time taken to separate the glass slides completely from each other [38].

#### 4.6.2. Keratin and Genistein Contents

In each formulation, the drug content was estimated by taking 0.1 mL of sample in a 10 mL volumetric flask and making up the volume with double-distilled water to prepare a stock solution. Further dilutions were prepared and analysed spectrophotometrically at λmax 222 nm and 280 nm, respectively. The experiment was performed in a triplicate manner.

#### 4.6.3. HPTLC

A previously discussed method, as stated above, with the mobile phase in butanol: acetic acid: water in the ratio of 4:1:1 *v*/*v*/*v* for keratin gel and chloroform: methanol in the ratio of 10:1 *v/v* for genistein gel was followed.

#### 4.6.4. Short-Term Stability Study

The formulation was subjected to accelerated conditions of temperature (40 ± 2 °C) and humidity (75 ± 5%) for the duration of 90 days. The formulations were packed in aluminium foil and kept inside the PVC bottle. After the completion of the study, the essential physical parameters and drug content were determined at pre-determined intervals of 30 days, 60 days, and, finally, 90 days. The obtained observations/parameters were then compared with the pharmacopoeia guidelines.

### 4.7. Animals

Male albino rats [(n = 30) (age: 5 to 6 weeks; weight range: 200–300g)] were randomly divided into five groups. There were four hydrogel-treated experimental groups, including (1) feather keratin hydrogel, (2) genistein hydrogel, (3) keratin–genistein hydrogel, and (4) marketed preparation, while the fifth group was a non-treated control group (six rats in each group) after obtaining animal study permission from the institutional animal ethical committee (IAEC) as per the Committee for Control and Supervision of Experiments on Animals (CCSEA) guidelines and histopathological studies with approval numbers 1426/PO/Re/S/11/CPCSEA. The animals were kept in the animal house, provided free access to water, also fed standard food, and maintained under a controlled environment (temperature 22 ± 2 °C; humidity 50–60% RH; and 12/12 h light and dark conditions) with proper hygiene. All animals were housed under standard environmental conditions to prevent any significant antibacterial interference with the wound due to external factors [39,40,41].

### 4.8. Wound Healing Study

For evaluating the wound-healing properties of the feather keratin, genistein, and keratin–genistein hydrogel, and marketed preparation, a full-thickness excision model was applied for the evaluation. All animals were anaesthetised with an intra-peritoneal ketamine injection (100 mg/mL) and xylazine, ilium-xylazine-20 (20 mg/mL). For anaesthetic administration, 7.5 mL of ketamine and 5 mL of xylazine were diluted with 7.5 mL of double-distilled water. A dosage of 0.2 mL/100 g body weight was used for the induction of complete anaesthesia. The dorsal hair was removed with a hair-removing cream (Anne French, Ahmedabad, Gujarat, India). A partial-thickness skin wound of dimension 1.5 × 1.5 cm was prepared via excision of the dorsal skin of the animal by using surgical scissors and forceps. Subsequently, the excised wound of the experimental group of rats was covered with the tested hydrogel (applied 1 fingertip unit (FTU)), and the bare wound was kept as a negative control. The probable changes observed in the wound area with time progression were measured by using a method of transparency-based digital imaging. On the 7th and 14th postoperative days, the reconstructed skins of the wounds were excised and fixed in 4% paraformaldehyde for histological observations and collagen deposition determination. At the selected post-wound intervals, photographs were taken. The wound closure was estimated according to the following equation [42,43]:

Wound closure (%) = [A_0_ − A_t_]/A_0_ × 100

where A_0_ is the initial wound area and at is the wound area at the same time interval of “t” days.

### 4.9. Biochemical Analysis

#### 4.9.1. Estimation of Cytokines by ELISA

Enzyme-Linked Immunosorbent Assay (ELISA) was performed to determine the cytokine levels of IL-1β, IL-2, IL-6, and IL-10 in liver tissue as per the manufacturer’s instructions [44].

#### 4.9.2. qRT-PCR Analysis

In total, 10 mg of tissue samples from all the groups was used to isolate total mRNA via the Triazole technique in order to examine the amount of mRNA that is expressed for the gene of interest. The RNeasy small kit was used to purify the mRNA as per the manufacturer’s instructions. Finally, qRT-PCR (Real-Time Quantitative Reverse Transcription PCR) was performed via the standard instrument with the help of the PCR master mix. Denaturation of the cDNA was performed at 94 °C for 5 min, followed by annealing at 58 °C for 30 s and subsequent extension at 72 °C for 35 s. Forty times repetition of the cycle was set by using qRT-PCR, which helps in the detection of the amplified DNA in real-time. To normalise mRNA, GAPDH was used as a housekeeping reference. For all the treated groups, ΔCt values was normalised with untreated control samples (ΔCt = Ct gene of interest − Ct housekeeping gene). The relative changes in the expression level of a particular gene were measured in terms of 2−ΔΔCt (ΔΔCt = ΔCt test − ΔCt control) [45,46].

### 4.10. Histopathological Investigations

The repaired skin of the wounds was taken out and preserved in a 4% paraformaldehyde solution on the 7th and 14th day following surgery for histopathological examination and to determine where collagen was deposited. To check the biocompatibility of biological macromolecules, viz., keratin and genistein, major tissues such as the liver and kidney were resected and fixed in 10% formalin for histological observations with respect to major changes associated with the tissue toxicity study.

Via selective staining with the dye haematoxylin and eosin, the histological analyses of wound skin, liver, and kidney were performed. The 7 mm to 10 mm thick sections were cut and initially fixed in formal calcium and embedded in paraffin wax. By employing xylene, the cut sections were de-waxed, treated with decreasing amounts of alcohol for hydration, and stained with haematoxylin. Then, the sections were dehydrated with alcohol to 70% concentration and further stained with 1% alcoholic eosin solution. Subsequently, the sections were differentiated in 90% alcohol solution, cleaned with xylene, mounted cautiously, and observed under the microscope [47,48].

### 4.11. Digital Image Analysis

From the histological images of tissues, luminescence was identified and transformed into the heights of the final 3D, interactive surface plot, which recognises the pure stained areas adjacent to the adjoining sampling in a square dimension and represents the background stains, nuclear sites, and other characteristics. A computerised scoring was allocated by examining and determining the staining pattern by using the Image J^®^ program.

### 4.12. Statistical Analysis

All values were provided as means with standard deviations (±SD), with n = 6; ** represented significance at *p* < 0.01 versus the control group, and * represented significance at *p* < 0.05 in comparison with the standard gel and keratin–genistein combination groups. By using Statistica^®^ v.17.0 software, one-way ANOVA was used to analyse the data, and then Bonferroni’s Multiple Comparison Test was performed.

## Figures and Tables

**Table 1:** Amino acid profiling of extracted keratin.

Test Parameter	Test Unit (g/100 g)	Reference Range (g/100 g)
Aspartic Acid	2. 3.15	3. 4.70
4. Threonine	5. 2.81	6. 4.10
7. Serine	8. 12.10	9. 13.57
10. Alanine	11. 2.41	12. 3.64
13. Glutamic Acid	14. 5.22	15. 9.10
16. Tyrosine	17. 0.36	18. 1.84

**Table 2:** HPTLC interpretation of various gel formulations.

Peak	Start Position (Rf)	Start Height (AU)	Max Position (Rf)	Max Height (AU)	Max %	End Position (Rf)	End Height (AU)	Area (AU)	Area %
**Keratin extract and keratin gel formulation**									
3	0.62	6.5	0.63	12.8	2.52	0.64	0.4	90.3	0.57
7	0.62	7.7	0.63	18.9	3.54	0.65	6.4	159.5	0.95
**Genistein extract and genistein gel formulation**									
6	0.56	42.9	0.56	49.2	8.81	0.60	38.7	802.0	11.03
5	0.48	26.8	0.50	41.4	13.37	0.51	25.6	448.2	13.34
**Keratin, genistein extract and keratin, genistein combination gel** **(Mobile Phase: butanol: acetic acid: water)**									
10	0.56	3.5	0.58	14.3	2.33	0.60	9.8	175.0	1.46
9	0.49	5.9	0.51	13.0	2.35	0.55	1.2	135.9	1.78
**Keratin, genistein extract and keratin, genistein combination gel** **(Mobile Phase: chloroform: methanol)**									
4	0.73	21.3	0.74	27.7	6.09	0.75	19.5	279.2	3.68
8	0.68	15.2	0.70	25.4	7.02	0.72	14.2	271.8	4.87

**Table 3:** Post-stability characterisation of gel formulations.

Gel Type	Gel Strength (Sec)	Drug Content Mean ± SD	pH Mean ± SD		Viscosity (Cps) Mean ± SD		Spreadability (gm·cm/s) Mean ± SD	
			Pre-Stability	Post-Stability	Pre-Stability	Post-Stability	Pre-Stability	Post-Stability
Keratin gel	4	96.07 ± 0.7214	7.6 ± 0.15	6.8 ± 0.15	470,913 ± 1978.7	433,342 ± 9495.1	9.78 ± 1.0	8.4 ± 0.85
Genistein gel	3	91.89 ± 0.2134	6.8 ± 0.1	6.4 ± 0.20	738,063 ± 2411.4	706,920 ± 1126.8	14 ± 1.4	13.65 ± 0.63
Keratin–Genistein gel	4	93.21 ± 0.764 (Keratin) 92.79 ± 0.7543 (Genistein)	7.2 ± 0.2	6.5 ± 0.26	714,547.53 ± 3040.3	700,446 ± 6777.86	12.56 ± 0.92	10.83 ± 1.2

**Table 4:** Short-term (90 days) stability studies of gel formulations.

Formulation	pH Mean ± SD		Viscosity (cPs) Mean ± SD		Spreadability (gm·cm/s) Mean ± SD	
	0 Days	90 Days	0 Days	90 Days	0 Days	90 Days
Keratin gel	7.6 ± 0.15	6.8 ± 0.15	470,913 ± 19,787.5	433,342 ± 9495.15	9.78 ± 1.0	8.4 ± 0.85
Genistein gel	6.8 ± 0.1	6.4 ± 0.20	738,063 ± 24,114.7	706,920 ± 11,268.6	14 ± 1.4	13.65 ± 0.63
Keratin-Genistein gel	7.2 ± 0.2	6.5 ± 0.26	714,547.53 ± 30,403.9	700,446 ± 6777.86	12.56 ± 0.92	10.83 ± 1.2

**Table 5:** Wound healing after 7 days and 14 days.

Groups	Day 7 (% Wound Healing) Mean ± SD	Day 14 (% Wound Healing) Mean ± SD
Keratin gel	54.29 ± 4.170	87.57 ± 5.00
Genistein gel	48.51 ± 7.88	87.40 ± 3.57
Keratin and genistein combination gel	51.18 ± 2.043	94.65 ± 4.64
Standard gel	54.99 ± 13.63	96.66 ± 5.77
Control	48.15 ± 9.32	85.98 ± 3.53

**Table 6:** ELISA measurement of interleukins (IL-2, IL-6, IL-1β, and IL-10) and COX-2 levels in liver after the application of keratin, genistein, and keratin–genistein gel.

Parameters	NC	WC	PC	Keratin	Genistein	Keratin and Genistein
COX-2 (pg/mL)	188.78 ± 8.09	449.24 ± 12.95	210.84 ± 10.51 ***	240.97 ± 8.46 ***	223.79 ± 11.49 ***	196.25 ± 9.61 ***
IL-2 (pg/mL)	492.27 ± 12.03	1103.32 ± 19.37	594.38 ± 12.45 ***	620.31 ± 16.85 ***	601.76 ± 18.17 ***	519.37 ± 11.51 ***
IL-6 (pg/mL)	148.91 ± 4.61	327.94 ± 8.16	179.24 ± 5.34 ***	204.37 ± 9.17 ***	191.38 ± 7.42 ***	163.84 ± 7.19 ***
IL-10 (pg/mL)	630.94 ± 20.37	1294.01 ± 25.79	764.85 ± 23.14 ***	804.59 ± 22.67 ***	789.41 ± 11.64 ***	681.93 ± 9.43 ***
IL1-β (pg/mL)	552.37 ± 18.19	1109.86 ± 24.17	624.87 ± 12.37 ***	655.49 ± 11.27 ***	634.59 ± 9.73 ***	611.48 ± 10.70 ***

Legends: NC: Normal Control; WC: Working Control; PC: Positive Control. *** Represents the level of significance in comparision with normal control/working control.

**Table 7:** Composition of keratin gel, genistein gel, and keratin–genistein gel (50 g quantity).

2% KERATIN GEL		1% GENISTEIN GEL		KERATIN–GENISTEIN GEL	
Ingredients	Quantity	Ingredients	Quantity	Ingredients	Quantity
Carbopol 934	1% *w*/*v*	Carbopol 934	1% *w*/*v*	Carbopol 934	1% *w*/*v*
Keratin	1 g	Genistein	0.5 g	Keratin	1 g
Cremophor RH 40	3 mL	Methyl paraben	0.01 g	Genistein	0.5 g
Methyl Paraben	0.01 g	Propyl paraben	0.01 g	Cremophor RH 40	3 mL
Propyl Paraben	0.01 g	Triethanolamine	q.s.	Methyl paraben	0.01 g
Triethanolamine	q.s.			Propyl paraben	0.01 g
				Triethanolamine	q.s.

## Data Availability

Not applicable.

## Abbreviations

TLC	Thin-Layer Chromatography
HPTLC	High-Performance Thin-Layer Chromatography
FTIR	Fourier-Transform Infrared Spectroscopy
SEM	Scanning Electron Microscopy
ELISA	Enzyme-Linked Immunoassay
qRT-PCR	Real-Time Quantitative Reverse Transcription PCR
IAEC	Institutional Animal Ethical Committee
CCSEA	Committee for Control and Supervision of Experiments on Animals
T-BHP	t-butylhydroperoxide
COX-2	Cyclooxygenase-2
IPQC	In-Process Quality Control

## References

[1] Guest J.F., Vowden K., Vowden P. (2017). The health economic burden that acute and chronic wounds impose on an average clinical commissioning group/health board in the UK. J. Wound Care.

[2] Cheng Q., Gibb M., Graves N., Finlayson K., Pacella R.E. (2018). Cost-effectiveness analysis of guideline-based optimal care for venous leg ulcers in Australia. BMC Health Serv. Res..

[3] Olsson M., Järbrink K., Divakar U., Bajpai R., Upton Z., Schmidtchen A., Car J. (2019). The humanistic and economic burden of chronic wounds: A systematic review. Wound Repair Regen..

[4] Gray T.A., Rhodes S., Atkinson R., Rothwell K., Wilson P., Dumville J.C., Cullum N. (2018). Opportunities for better value wound care: A multiservice, cross-sectional survey of complex wounds and their care in a UK community population. BMJ Open.

[5] Casado-Díaz A., La Torre M., Priego-Capote F., Verdú-Soriano J., Lázaro-Martínez J.L., Rodríguez-Mañas L., Pérez M.B., Tunez I. (2022). EHO-85: A Multifunctional Amorphous Hydrogel for Wound Healing Containing *Olea europaea* Leaf Extract: Effects on Wound Microenvironment and Preclinical Evaluation. J. Clin. Med..

[6] Pastar I., Stojadinovic O., Yin N.C., Ramirez H., Nusbaum A.G., Sawaya A., Patel S.B., Khalid L., Isseroff R.R., Tomic-Canic M. (2014). Epithelialization in Wound Healing: A Comprehensive Review. Adv. Wound Care.

[7] Boateng J.S., Matthews K.H., Stevens H.N., Eccleston G.M. (2008). Wound Healing Dressings and Drug Delivery Systems: A Review. J. Pharm. Sci..

[8] Chattopadhyay N., Zastre J., Wong H.-L., Wu X.Y., Bendayan R. (2008). Solid Lipid Nanoparticles Enhance the Delivery of the HIV Protease Inhibitor, Atazanavir, by a Human Brain Endothelial Cell Line. Pharm. Res..

[9] Rouse J.G., Van Dyke M.E. (2010). A Review of Keratin-Based Biomaterials for Biomedical Applications. Materials.

[10] Khosa M.A., Ullah A.J.J.F.P. (2013). A sustainable role of keratin biopolymer in green chemistry: A review. J. Food Process. Beverages.

[11] Hill P., Brantley H., Van Dyke M. (2010). Some properties of keratin biomaterials: Kerateines. Biomaterials.

[12] Kumaran P., Gupta A., Sharma S. (2017). Synthesis of wound-healing keratin hydrogels using chicken feathers proteins and its properties. Int. J. Pharm. Pharm. Sci..

[13] Ar B., Choudhury K. (2018). Study on the Effect of Genistein, a Soy Isoflavone in Insulin Tolerance in Albino Rat (*Rattus albicans*). Sch. Acad. J. Biosci..

[14] Emmerson E., Campbell L., Ashcroft G.S., Hardman M.J. (2010). The phytoestrogen genistein promotes wound healing by multiple independent mechanisms. Mol. Cell. Endocrinol..

[15] Duchnik E., Kruk J., Baranowska-Bosiacka I., Pilutin A., Maleszka R., Marchlewicz M. (2019). Effects of the soy isoflavones, genistein and daidzein, on male rats’ skin. Adv. Dermatol. Allergol..

[16] Marini H.R., Polito F., Altavilla D., Irrera N., Minutoli L., Calò M., Adamo E.B., Vaccaro M., Squadrito F., Bitto A. (2010). Genistein aglycone improves skin repair in an incisional model of wound healing: A comparison with raloxifene and oestradiol in ovariectomized rats. Br. J. Pharmacol..

[17] Emmerson E., Hardman M.J. (2012). The role of estrogen deficiency in skin ageing and wound healing. Biogerontology.

[18] Rohrmann S., Shvetsov Y.B., Morimoto Y., Wilkens L.R., Monroe K.R., Le Marchand L., Franke A.A., Kolonel L.N., Maskarinec G. (2018). Self-reported dietary flavonoid intake and serum markers of inflammation: The multiethnic cohort. Cancer Causes Control.

[19] Irrera N., Pizzino G., D’anna R., Vaccaro M., Arcoraci V., Squadrito F., Altavilla D., Bitto A. (2017). Dietary Management of Skin Health: The Role of Genistein. Nutrients.

[20] Hwang K., Chung R.S., Schmitt J.M., Buck D., Winn S.R., Hollinger J.O. (2001). The Effect of Topical Genistein on Soft Tissue Wound Healing in Rats. J. Histotechnol..

[21] Cooke P.S., Selvaraj V., Yellayi S. (2006). Genistein, estrogen receptors, and the acquired immune response. J. Nutr..

[22] Sinkiewicz I., Śliwińska A., Staroszczyk H., Kołodziejska I. (2017). Alternative Methods of Preparation of Soluble Keratin from Chicken Feathers. Waste Biomass Valorization.

[23] Sadowska-Krowicka H., Mannick E.E., Oliver P.D., Sandoval M., Zhang X.-J., Eloby-Childess S., Clark D.A., Miller M.J.S. (1998). Genistein and Gut Inflammation: Role of Nitric Oxide. Proc. Soc. Exp. Boil. Med..

[24] Kharwade R., Ali N., Gangane P., Pawar K., More S., Iqbal M. (2023). DOE-Assisted Formulation, Optimization, and Characterization of Tioconazole-Loaded Transferosomal Hydrogel for the Effective Treatment of Atopic Dermatitis: In Vitro and In Vivo Evaluation. Gels.

[25] Seibel J., Molzberger A.F., Hertrampf T., Laudenbach-Leschowski U., Diel P. (2009). Oral treatment with genistein reduces the expression of molecular and biochemical markers of inflammation in a rat model of chronic TNBS-induced colitis. Eur. J. Nutr..

[26] Kharwade R.S., Mahajan N.M. (2019). Formulation and Evaluation of Nanostructured Lipid Carriers Based Anti-Inflammatory Gel for Topical Drug Delivery System. Asian J. Pharm. Clin. Res..

[27] Tie L., An Y., Han J., Xiao Y., Xiaokaiti Y., Fan S., Liu S., Chen A.F., Li X. (2013). Genistein accelerates refractory wound healing by suppressing superoxide and FoxO1/iNOS pathway in type 1 diabetes. J. Nutr. Biochem..

[28] Stipcevic T., Piljac A., Piljac G. (2006). Enhanced healing of full-thickness burn wounds using di-rhamnolipid. Burns.

[29] Woods A. (2001). Syndecans: Transmembrane modulators of adhesion and matrix assembly. J. Clin. Investig..

[30] Greene D.K., Tumova S., Couchman J.R., Woods A. (2003). Syndecan-4 Associates with α-Actinin. J. Biol. Chem..

[31] Diegelmann R.F., Cohen I.K., Kaplan A.M. (1981). The Role of Macrophages in Wound Repair: A review. Plast. Reconstr. Surg..

[32] Gupta A., Perumal R., Yunus R.B.M., Kamarudin N.B. (2011). Extraction of keratin protein from chicken feather. J. Chem. Chem. Eng..

[33] Šafarič R., Zemljič L.F., Novak M., Dugonik B., Bratina B., Gubeljak N., Bolka S., Strnad S. (2020). Preparation and Characterisation of Waste Poultry Feathers Composite Fibreboards. Materials.

[34] Wang H., Pampati N., McCormick W.M., Bhattacharyya L. (2016). Protein Nitrogen Determination by Kjeldahl Digestion and Ion Chromatography. J. Pharm. Sci..

[35] Fekkes D., van Dalen A., Edelman M., Voskuilen A. (1995). Validation of the determination of amino acids in plasma by high-performance liquid chromatography using automated pre-column derivatization with o-phthaldialdehyde. J. Chromatogr. B Biomed. Sci. Appl..

[36] Blackburn S., Lowther A.G. (1951). The action of organic acids on some fibrous proteins: The oxidation of wool keratin. Biochem. J..

[37] Elhawary Y., Badria F. (2011). Mode of Action of Centella Asiatica in Wound Healing Using Immunohistochemical Studies. Egypt. Dent. J..

[38] Ullah N., Amin A., Farid A., Selim S., Rashid S.A., Aziz M.I., Kamran S.H., Khan M.A., Khan N.R., Mashal S. (2023). Development and Evaluation of Essential Oil-Based Nanoemulgel Formulation for the Treatment of Oral Bacterial Infections. Gels.

[39] Jia Z., Babu P.V.A., Si H., Nallasamy P., Zhu H., Zhen W., Misra H.P., Li Y., Liu D. (2013). Genistein inhibits TNF-α-induced endothelial inflammation through the protein kinase pathway A and improves vascular inflammation in C57BL/6 mice. Int. J. Cardiol..

[40] Fule R., Kaleem M., Asar T.O., Rashid A., Shaik R.A., Eid B.G., Nasrullah M.Z., Ahmad A., Kazmi I. (2023). Formulation, Optimization and Evaluation of Cytarabine-Loaded Iron Oxide Nanoparticles: From In Vitro to In Vivo Evaluation of Anticancer Activity. Nanomaterials.

[41] Omer A.B., Dalhat M.H., Khan M.K., Afzal O., Altamimi A.S.A., Alzarea S.I., Almalki W.H., Kazmi I. (2022). Butin Mitigates Memory Impairment in Streptozotocin-Induced Diabetic Rats by Inhibiting Oxidative Stress and Inflammatory Responses. Metabolites.

[42] Bahramsoltani R., Farzaei M.H., Rahimi R. (2014). Medicinal plants and their natural components as future drugs for the treatment of burn wounds: An integrative review. Arch. Dermatol. Res..

[43] Midwood K.S., Williams L.V., Schwarzbauer J.E. (2004). Tissue repair and the dynamics of the extracellular matrix. Int. J. Biochem. Cell Biol..

[44] Singh A.K., Bhadauria A.S., Kumar U., Raj V., Rai A., Kumar P., Keshari A.K., Kumar D., Maity B., Nath S. (2018). Novel Indole-fused benzo-oxazepines (IFBOs) inhibit invasion of hepatocellular carcinoma by targeting IL-6 mediated JAK2/STAT3 oncogenic signals. Sci. Rep..

[45] Park E., Lee S.M., Jung I.-K., Lim Y., Kim J.-H. (2011). Effects of genistein on early-stage cutaneous wound healing. Biochem. Biophys. Res. Commun..

[46] Alghamdi R.M., Hassan M.A., Kaleem M., Kayali A., Halwani M.A., Zamzami M.A., Choudhry H., Alhosin M. (2020). Targeting Itch/p73 pathway by thymoquinone as a novel therapeutic strategy for cancers with p53 mutation. Eur. J. Cell Sci..

[47] Al Khalaf A.K., Abdulrahman A.O., Kaleem M., Nur S.M., Asseri A.H., Choudhry H., Khan M.I. (2021). Comparative Analysis of the Impact of Urolithins on the Composition of the Gut Microbiota in Normal-Diet Fed Rats. Nutrients.

[48] Kaleem M., Haque S.E. (2015). Evaluation of Cardioprotective Role of Vinpocetine in Isoproterenolinduced Myocardial Infarction in Rats. J. Pharm. Res..

